# Effectiveness of various irrigation protocols for the removal of calcium hydroxide from artificial standardized grooves

**DOI:** 10.1590/1678-7757-2016-0414

**Published:** 2017

**Authors:** Hakan GOKTURK, Ismail OZKOCAK, Feyzi BUYUKGEBIZ, Osman DEMIR

**Affiliations:** 1 Gaziosmanpasa University, Faculty of Dentistry, Department of Endodontics, Tokat, Turkey.; 2 Gaziosmanpasa University, Faculty of Medicine, Department of Biostatistics, Tokat, Turkey.

**Keywords:** Calcium hydroxide, Endodontics, Lasers, Sodium hypochlorite, Ultrasonic therapy

## Abstract

**Objective:**

The aim of this study was to investigate the ability of laser-activated irrigation (LAI), XP-endo Finisher, CanalBrush, Vibringe, passive ultrasonic irrigation (PUI), and conventional syringe irrigation systems on the removal of calcium hydroxide (CH) from simulated root canal irregularities.

**Material and Methods:**

The root canals of one hundred and five extracted single-rooted teeth were instrumented using Reciproc rotary files up to size R40. The teeth were split longitudinally. Two of the three standard grooves were created in the coronal and apical section of one segment, and another in the middle part of the second segment. The standardized grooves were filled with CH and the root halves were reassembled. After 14 days, the specimens were randomly divided into 7 experimental groups (n=15/group). CH was removed as follows: Group 1: beveled needle irrigation; Group 2: double side-vented needle irrigation; Group 3: CanalBrush; Group 4: XP-endo Finisher; Group 5: Vibringe; Group 6: PUI; Group 7: LAI. The amount of remaining CH in the grooves was scored under a stereomicroscope at 20× magnification. Statistical evaluation was performed using Kruskal–Wallis and Bonferroni-Correction Mann–Whitney U tests.

**Results:**

Groups 1 and 2 were the least efficient in eliminating CH from the grooves. Groups 6 and 7 eliminated more CH than the other protocols; however, no significant differences were found between these two groups (P>.05).

**Conclusions:**

Nevertheless, none of the investigated protocols were able to completely remove all CH from all three root regions. LAI and PUI showed less residual CH than the other protocols from artificial grooves.

## Introduction

Chemomechanical preparation is the first step to eliminate microorganisms in the root canal system, but it alone is not sufficient to clean the root canal. *Ex vivo* and clinical studies have indicated that intact areas remain on root canal walls during mechanical preparation, and therefore, it is important to perform irrigation in addition to mechanical preparation^15,23^. For this purpose, several different irrigation solutions, medicaments and techniques have been used^10,11,14^.

Calcium hydroxide (CH) is widely used as an intracanal medicament between appointments to increase the number of canals free from bacteria because of its antibacterial, therapeutic, biocompatible, and regenerative properties^4,26^. However, the remnant CH hinders the penetration of disinfectants and sealers into dentinal tubules and compromises the seal of the canal filling^17,22^. Therefore, residual CH must be removed before permanent root canal obturation is completed^8^. In most cases, the residual CH was removed following copious irrigation with sodium hypochlorite (NaOCl) and ethylenediaminetetraacetic acid (EDTA) in combination with the use of master apical file (MAF) or gutta-percha up to working length (WL)^24^. Different irrigation techniques and devices were used to activate and improve the effectiveness of irrigation solutions^7,10,14,25^.

A novel irrigation instrument, the XP-endo Finisher, has been introduced by FKG, Dentaire SA (La Chaux-de-Fonds, Switzerland). This instrument’s design is similar to an ISO size #25, 0.00 taper NiTi file. According to manufactures, this file improves the penetration of irrigation solutions to the irregular area of root canal system by expanding its reach 6 mm in diameter^3,29^.

Although previous studies demonstrated efficacy of sonic and ultrasonic systems on debris and CH removal^8,10,28^, sufficient information is not yet available in the literature concerning XP-endo Finisher^31^ and laser-activated irrigation (LAI). Therefore, the aim of this study was to compare the effect of XP-endo Finisher, sonic, ultrasonic and LAI and conventional syringe irrigation techniques in removing CH from simulated lateral irregularities on the root canal wall. The null hypotheses were that the removal of CH was not affected by irrigation technique [1] or the [2] section of root canal (third).

## Material and methods

**Figure 1:**
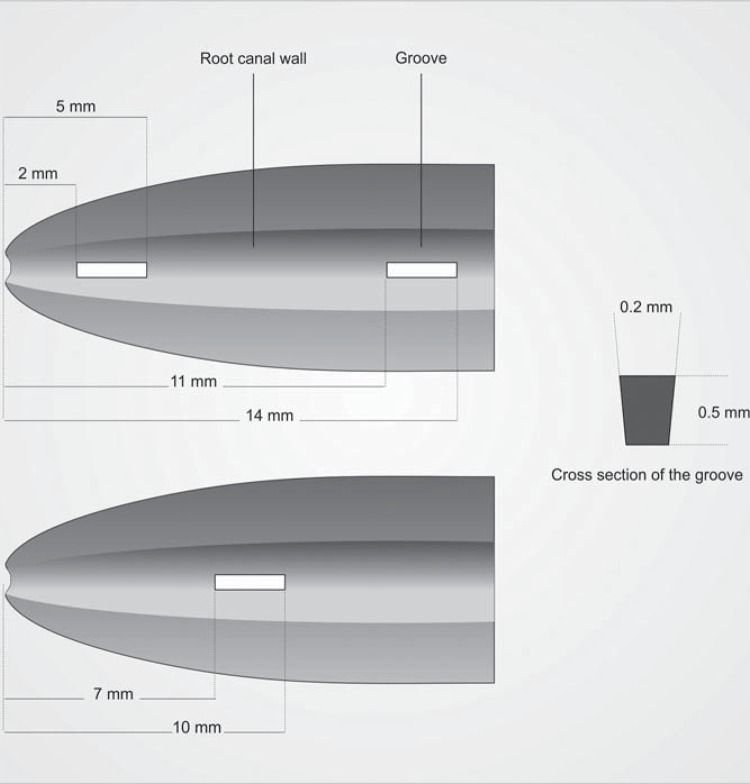
A schematic representation of the location and size of the longitudinal grooves

Ethical approval was obtained from the Clinical Research Ethics Committee of the Faculty of Medicine of Gaziosmanpasa University (project number: 15-KAEK-229). One hundred and five human maxillary incisors were used in this study. Mesiodistal and bucco-palatal direction radiographs were taken from the teeth to confirm the presence of a single canal. The teeth were decoronated using straight diamond burs (Komet, Gebr. Brasseler GmbH & Co. KG, Germany) in a conventional high-speed handpiece under water cooling so that each root had a standardized length of 15 mm. The WL was determined to be 1 mm short of the apex using a #10 K file (VDW GmbH, Munich, Germany). The WL was established at 14 mm. Roots were prepared with Reciproc rotary files up to size R40 at WL (VDW GmbH, Germany) and irrigation was performed with 10 mL 2.5% NaOCl. Next, roots were placed in Eppendorf tubes (Labosel, İstanbul, Turkey) filled with a silicone material (Zetaplus soft; Zhermack Clinical, Badia Polesine, Italy). After removal of the roots from the impression material, a longitudinal groove was prepared on the buccal and lingual surface using a narrow diamond bur without cutting the canal wall. A spatula was used to split longitudinally. A number 1S cavitron tip (Aceton, Merignac, France) was modified and inserted into an ultrasonic handpiece (Newtron P5; Satelec, Acteongroup, France) to create artificial standardized grooves. Two of the three standard grooves were created in the coronal and apical part of one segment, and another in the middle part of a second segment. The dimensions of grooves were 0.2 mm in width, 3 mm in length, and 0.5 mm in depth (Figure 1). The dimension of grooves was checked under a stereomicroscope (Zeiss Stemi 2000-C, Carl Zeiss MicroImaging, Göttingen, Germany) at 20X magnification. The root halves and grooves were irrigated with 5 mL of 17% EDTA for 1 min. and 5 mL 2.5% NaOCl for 1 min. while being activated with a toothbrush to remove debris and the smear layer. The standardized grooves were filled with CH paste (Ammdent, Punjab, India), and the root halves were reassembled. The root canals were fully filled with CH paste using a Lentulo spiral. Two radiographs (mesiodistal and bucco-palatal direction) were taken to confirm complete filling of the canals with CH paste. Roots were remounted and placed into Eppendorf tubes. Then samples were divided randomly into 7 groups, each containing 15 teeth. Seven different color stickers were pasted on the caps of the Eppendorf tubes to indicate each of the 7 groups. The access cavities were sealed with temporary filling material (Cavit, 3M ESPE, Seefeld, Germany) and stored in 37°C at 100% relative humidity for 2 weeks.

After 14 days, the CH was removed as follows: first, an R40 file (VDW, Germany) at WL and 1 mL 2.5% NaOCl was used to obtain a space for irrigation needles and instruments.

Group 1- Beveled needle irrigation: The root canals were irrigated with 10 mL 2.5% NaOCl for 2 min. via 27-gauge beveled dental irrigation needle (Ayset, Adana, Turkey). The needle tip was placed 1 mm short of the WL.

Group 2- Double side-vented needle irrigation: The root canals were irrigated with 10 mL 2.5% NaOCl for 2 min. via a 30-gauge double side-vented needle (i-Tips, i dental, Siauliai, Lithuania). The needle tip was placed 1 mm short of the WL.

Group 3- CanalBrush: The root canals were irrigated with 5 mL 2.5% NaOCl and then brushed with a medium size CanalBrush (Coltene/Whaledent GmbHCo. KG, Langenau, Germany) at 600 rpm for 1 min. A final flush was done with 5 mL 2.5% NaOCl. CanalBrushes were inserted 1 mm short of the WL and moved with small vertical movements.

Group 4- XP-endo Finisher: Irrigation protocol was same as Group 3 with the exception that the XP-endo Finisher (FKG, Dentaire Sa, La Chaux-de- Fonds, Switzerland) was used instead of the CanalBrush at 800 rpm with 1 Ncm for 1 min. The file tip was placed 1 mm short of the WL.

Group 5- Sonic Irrigation (Vibringe): A 10 mL 2.5% NaOCl was delivered and sonically activated via the Vibringe system (Vibringe B. V. Corp, Amsterdam, Netherlands). The needle tip was placed 1 mm short of the WL without touching the canal walls, enabling it to vibrate freely for 2 min.

Group 6- Passive ultrasonic irrigation (PUI): Irrigation protocol was the same as Group 3, with the exception that the passive ultrasonic activation was performed using an Irrisafe ultrasonic tip (size 25, 0.02 taper) (Satelec Acteongroup, France) that was placed 1 mm short of the WL. A power setting of 9 was used for duration of 1 min. A 10 mL 2.5% NaOCl solution continuously delivered at a flow rate of approximately 0.16 mL s^-1^through the unit.

Group 7- Er:YAG laser-activated irrigation: The irrigation solution was activated with the same protocol as in Group 3, with the exception that an Er:YAG laser (Kavo Key 3+, KaVo, Biberach, Germany) with a 2940 nm wavelength for 1 min. was used with endodontic tips (a 28 mm long and diameter ISO 30) in place of a CanalBrush. Laser parameters were 1 W, 10 Hz, 100 mJ, and an energy density of 142.8 J/cm^2^. The laser tip was inserted into canal at 1 mm short of the WL. When the root canal irrigant dropped or vaporized, the canal space was filled with 2.5% NaOCl.

**Figure 2:**
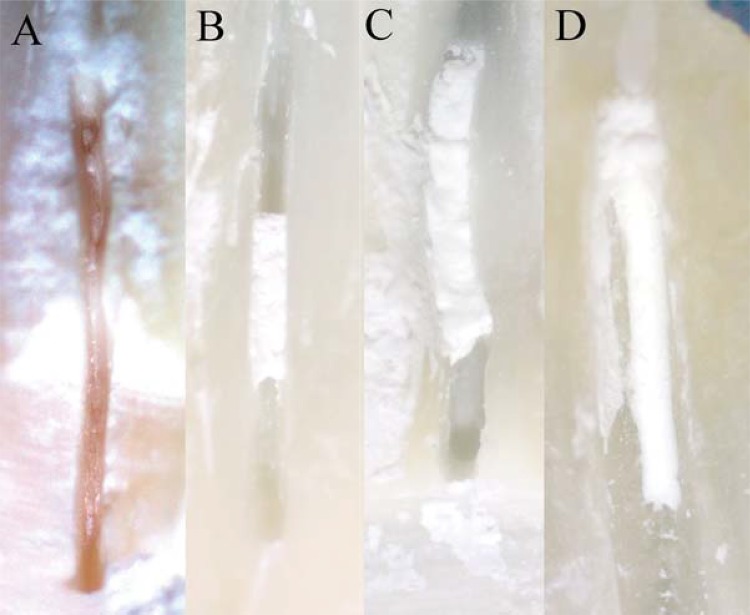
Scores of Ca(OH)2 remnants: (A) Score 0 (group 7 at apical region); (B) Score 1 (group 6 at middle region); (C) Score 2 (group 5 at coronal region); (D) Score 3 (group 1 at apical region)

For each specimen, 11 mL of 2.5% NaOCl was used as irrigation solution and was delivered at a flow rate of approximately 0.08 mL s-1 except for G6 (PUI). The irrigant was delivered into the canal with a double side-vented needle (i dental, Lithuania) except for Group 1 (beveled needle). The amount of remaining CH in the grooves was evaluated under a stereomicroscope (Zeiss Stemi 2000-C, Germany) at 20× magnification and equipped with a digital camera (AxioCam ERc5s, Germany) by two calibrated endodontists using an numeric evaluation scale described by van der Sluis, et al.^30^ (2007). The scoring system was as follows: score 0, the groove is entirely empty; score 1, CH is present in less than 50% of the groove; score 2, CH is present in more than 50% of the groove, but not completely; and score 3, the groove is completely covered with CH (Figure 2). Evaluation was conducted based on the color codes by two endodontists blinded to the group number. Before scoring, the two endodontists assessed 50 randomly selected specimens simultaneously for calibration purposes. In the case of discrepant scores, a consensus was reached by discussion.

Kruskal-Wallis test was used to compare the non-normal data among groups. For multiple comparisons between the pair-wise groups, Bonferroni-Correction Mann Whitney U test was used. A *p*-value <.05 was considered significant. The kappa coefficient was used to determine interexaminer agreement. Analyses were performed using SPSS 19 (IBM SPSS Statistics 19, SPSS Inc., Somers, NY).

## Results

Results of the two examiners were in good agreement (kappa value=0.897). Comparisons between the groups are presented in Tables 1 and 2. Elimination of CH was more difficult from the apical region. None of the irrigation protocols could completely remove all remnant of CH in all three root regions. Beveled needle irrigation (Group 1) and double side-vented needle irrigation (Group 2) were the significantly least efficient on the elimination of CH from the grooves (*P*<.001). PUI (Group 6) and Er:YAG laser-activated irrigation (Group 7) removed more CH than the other protocols in all thirds of the root; however, no significant differences were found between these two groups (*P*>.05). No significant differences were found between XP-endo Finisher (Group 4) and PUI (Group 6) at the coronal and middle regions (*P*>.05). The Kruskal-Wallis test showed significant differences between the groups for coronal, middle, and apical thirds (*P<*.05), except for the CanalBrush (Group 3) (*P*>.05).

**Table 1:** Multiple comparisons according to root canal regions

						Kruskal-Wallis statistical analysis	
Scores		0	1	2	3	Median[IQR]	P
Group 1 (Beveled Needle)	Coronal Middle Apical	- - -	- - -	8 5 1	7 10 14	2[2-3]^a^ 3[2-3]^ab^ 3[3-3]^b^	0.023
Group 2 (Double Side - Needle)	Coronal Middle Apical	- - -	-	10 5 2	5 10 13	2[2-3]^a^ 3[2-3]^ab^ 3[3-3]^b^	0.011
Group 3 (CanalBrush)	Coronal Middle Apical	- - -	- 3 1	14 9 7	1 3 7	2[2-2] 2[2-2] 2[2-3]	0.098
Group 4 (XP-endo Finisher	Coronal Middle Apical	- - -	4 4 -	11 9 7	- 2 8	2[1-2]^a^ 2[1-2]^a^ 3[2-3]^b^	0.001
Group 5 (Vibringe)	Coronal Middle Apical	- - -	2 1 -	13 12 8	- 2 7	2[2-2]^a^ 2[2-2]^ab^ 2[2-3]^b^	0.004
Group 6 (PUI)	Coronal Middle Apical	7 7 1	5 5 7	3 3 7	- - -	1[0-1]^a^ 1[0-1]^a^ 1[1-2]^b^	0.029
Group 7 (LAI)	Coronal Middle Apical	6 6 2	6 8 5	3 1 8	- - -	1[0-1]^ab^ 1[0-1]^a^ 2[1-2]^b^	0.022

**Table 2:** Multiple comparisons between groups

						Kruskal-Wallis statistical analysis	
Scores		0	1	2	3	Median[IQR]	P
Coronal	Beveled Needle	-	-	8	7	2[2-3]^ac^	<.001
	Double Side - Needle	-	-	10	5	2[2-3]^ac^	
	CanalBrush	-	-	14	1	2[2-2]^ac^	
	XP-endo Finisher	-	4	11	-	2[1-2]^bc^	
	Vibringe	-	2	13	-	2[2-2]^ac^	
	PUI	7	5	3	-	1[0-1]^b^	
	LAI	6	6	3	-	1[0-1]^b^	
Middle	Beveled Needle	-	-	5	10	3[2-3]^a^	<.001
	Double Side - Needle	-	-	5	10	3[2-3]^a^	
	CanalBrush	-	3	9	3	2[2-2]^a^	
	XP-endo Finisher	-	4	9	2	2[1-2]^ac^	
	Vibringe	-	1	12	2	2[2-2]^a^	
	PUI	7	5	3	-	1[0-1]^bc^	
	LAI	6	8	1	-	1[0-1]^b^	
Apical	Beveled Needle	-	-	1	14	3[3-3]^a^	<.001
	Double Side - Needle	-	-	2	13	3[3-3]^a^	
	CanalBrush	-	1	7	7	2[2-3]^a^	
	XP-endo Finisher	-	-	7	8	3[2-3]^a^	
	Vibringe	-	-	8	7	2[2-3]^a^	
	PUI	1	7	7	-	1[1-2]^b^	
	LAI	2	5	8	-	2[1-2]^b^	

## Discussion

Irrigation has an important role in controlling endodontic infection and debridement of the root canal system. CH is a widely used intracanal medicament as it creates a physical barrier from microorganisms and avoids the development of reinfections. However, previous studies have shown that removal of CH before the completion of the root canal obturation increased the sealer penetration into the dentinal tubules, and thus provided a good seal and also strengthens the bond between dentine and sealer^6,11^.

In the practice of endodontics, many different irrigation methods are used for this purpose. In this study, the effectiveness of different irrigation techniques on CH removal was evaluated. In the literature, calculation of the residual amount of CH remaining in the root canal were made by calculating the area of the remnant on dentin wall, by scoring, SEM analysis, volume analysis with spiral CT, and by using a micro-CT^5,25,30,32^. We preferred the scoring method described in the study by van der Sluis, et al.^30^ (2007) because it is a simple and easily accessible technique and used in many previous studies^1,8^.

CH powder was mixed with liquid and used in paste form in this study. Lambrianidis, et al.^18^ (1999) used CH medications at 42% and 95% concentrations in their study and reported that CH content in the paste had no effect on removal from the root canal wall.

A commonly suggested method for removal of CH is the irrigation with NaOCl and EDTA accompanied by very light instrumentation and the use of MAF^19,25^. Many techniques have been proposed to increase the efficiency of the irrigation solution. Mechanical agitation with a handpiece, gutta-percha or plastic tools, sonic and ultrasonic activation are also suggested techniques. In addition, LAI is another efficient and current method^13,21^. Kenee, et al.^16^ (2006) removed CH by four different procedures and demonstrated that the use of hand files and irrigation solution alone was not very effective on the removal of CH.

Capar, et al.^8^ (2014) compared CH removal efficiency of EndoVac, Self-Adjusting File (SAF), PUI and conventional irrigation techniques from artificial standardized grooves. They reported that PUI was more effective than other groups when NaOCl was used. Ahmetoglu, et al.^1^ (2013) compared CH removal efficiency of PUI, SAF and conventional irrigation methods by SEM. Researchers found that PUI was more effective than SAF and traditional irrigation. These results support our research. PUI technique is based on the transmission of acoustic energy to an irrigation solution. The agitation increases the penetration of irrigant to the irregular canal areas and the CH removal capacity of the irrigation solution. Similarly, van der Sluis, et al.^30^ (2007) investigated the efficiency of CH removal of various irrigation processes and found that PUI with NaOCl was more effective than PUI with water and syringe irrigation with NaOCl.

In this study, the effectiveness of different activation protocols of irrigation solutions were compared between themselves and with traditional irrigation methods. The results are consistent with the findings of the above-mentioned authors. According to our results, LAI and PUI were more effective in the elimination of CH in all 3 regions of the root canal. Therefore, the null hypothesis [1] that no differences would occur among the different irrigation techniques in terms of CH removal was rejected. In addition, no difference was found between PUI and XP-endo Finisher groups in the coronal and middle third regions. Although CanalBrush and sonic-activated irrigation exhibited lower scores than needle irrigation groups, the difference was not statistically significant. Results of our study indicate that there was a significant difference between sections of the root canal in terms of CH removal except for the CanalBrush group; therefore, the second null hypothesis is also rejected.

Despite results by Balvedi, et al.^5^ (2010), who stated PUI was more effective in terms of CH removal than syringe irrigation in the coronal and middle third regions, no statistically significant difference was found between the two irrigation regimes in the apical third. In the aforementioned study, researchers mixed CH with different liquids and preferred saline solution to the irrigant. In this study, CH was mixed with only distilled water and 2.5% NaOCl was used as the irrigation solution. The reason for the differences between the studies may be the various solutions and CH vehicle used. Similar to our findings, a recent systematic review showed the superiority of PUI over syringe irrigation on the removal of CH from the apical part of root canals^10^.

Wiseman, et al.^32^ (2011) evaluated the efficacy of CH removal with sonic irrigation and ultrasonic irrigation in the mesial root canal of mandibular molars by using the same volume irrigant (6% NaOCl+14% EDTA) and same time interval. Authors found PUI repeated 3 times with 20 sec. intervals was more effective than sonic irrigation. This study’s results are similar to the findings of Wiseman, et al.^32^ (2011), who found PUI was superior to sonic irrigation, although a different duration and irrigation regime was applied.

Tasdemir, et al.^27^ (2011) evaluated the use of NaOCl and NaOCl+EDTA with different agitation techniques (CanalBrush, PUI, MAF) on the removal of CH. Although the type of solution did not influence the CH removal, CanalBrush and PUI were found statistically more effective than other techniques. Our results were not consistent with the findings of the aforementioned study. Although CanalBrush removed more CH than the needle groups, this difference was not statistically significant. Another difference is that the PUI removed significantly more CH than CanalBrush in all three root regions. Tasdemir, et al.^27^ (2011) used field measurement methods as opposed to our study that utilized a preferred scoring method with a PUI time interval of 1 min.

Alturaiki, et al.^2^ (2015) compared the CH removal capacity of EndoVac, sonic, ultrasonic activated irrigation techniques with conventional irrigation. Researchers reported that no statistically significant difference was found between sonic and ultrasonic irrigation in the coronal and middle third regions, but found that sonic irrigation was more efficient in the apical region. Our results do not coincide with the results of these authors. The differences between this outcome and our results may reflect different variables in the study design, such as (i) the sonic device used and (ii) the MAF size. Researchers used EndoActivator (Dentsply Tulsa Dental Specialties, Tulsa, OK), NaOCl and EDTA in combination and prepared root canals up to #45 MAF. In this study, root canals were prepared up to #40 MAF and only 2.5% NaOCl was activated via Vibringe system. However, Bolles, et al.^7^ (2013) stated that no significant differences were found between EndoActivator and Vibringe systems on the sealer penetration into dentinal tubules. Further investigations are needed to clarify the effectiveness of these devices in removing medicaments.

Li, et al.^21^ (2015) compared CH removal efficacy of conventional needle irrigation, sonic, ultrasonic activated irrigation, and Photon-Induced Photoacoustic Streaming methods in root canals and isthmuses. Study results showed that laser and ultrasonic activated irrigation protocols were the most effective methods on the elimination of CH in both apical region and isthmuses. The findings of Li, et al.^21^ (2015) support the results of our research. According to our results, PUI was the most effective method for removal of CH than needle irrigation techniques in all three root regions. In addition, no statistically significant difference was found between XP-endo Finisher and PUI groups in the coronal and middle regions of root canals. However, Leoni, et al.^20^ (2016) stated that no significant differences were found between PUI and XP-endo Finisher in removing debris from root canal surface.

The XP-endo Finisher is newly introduced NiTi file to improve efficacy of final irrigation procedure. The canal must be shaped at least ISO 25 file to use XP-endo Finisher. The instrument is stabile in martensite form at room temperature and can be bent to the desired shape. The instrument changes its phase to austenite when the temperature reaches the body temperature^3,29^. We believe that only one study^31^ compared the effectiveness of the XP-endo Finisher on CH removal. In contrast to our findings, Wigler, et al.^31^ (2016) reported that no significant difference was found between PUI and XP-endo Finisher on the removal of CH from simulated irregularities in the apical third of root canals. However, previous studies showed its superior debris removal ability from the root canal walls^3,20^.

The apical third exhibited higher amounts of residual CH than the coronal and middle thirds in all experimental groups, except for LAI. This finding is in line with the results of previous studies^12,21^. This observation may be related to accumulation and transfer of residual CH to the apical region, which has smaller canal area and smaller volume of irrigation solution, as well as the anatomic complexity of apical third^9,12^. The action and circulation of irrigants may therefore be hindered.

## Conclusion

The activation of NaOCl with different instruments enhanced CH removal. Nevertheless, none of the investigated protocols were able to completely remove the CH from all three root regions. LAI and PUI methods removed more CH than the other protocols from artificial grooves in all thirds of the root canal.
